# A food systems approach and qualitative system dynamics model to reveal policy issues within the commercial broiler chicken system in South Africa

**DOI:** 10.1371/journal.pone.0270756

**Published:** 2022-06-29

**Authors:** Kevin Queenan, Soledad Cuevas, Tafadzwanashe Mabhaudhi, Michael Chimonyo, Bhavani Shankar, Rob Slotow, Barbara Häsler

**Affiliations:** 1 Department of Pathobiology and Population Sciences, Veterinary Epidemiology, Economics and Public Health (VEEPH) Research, Royal Veterinary College, Hertfordshire, United Kingdom; 2 Centre for Development, Environment and Policy, SOAS, London, United Kingdom; 3 Centre for Transformative Agricultural and Food Systems, School of Agricultural, Earth and Environmental Sciences, University of KwaZulu-Natal, Pietermaritzburg, South Africa; 4 International Water Management Institute (IWMI-GH), Southern Africa Office, Pretoria, South Africa; 5 School of Agricultural, Earth and Environmental Sciences, University of KwaZulu-Natal, Pietermaritzburg, South Africa; 6 Institute for Sustainable Food, University of Sheffield, Sheffield, United Kingdom; 7 School of Life Sciences, University of KwaZulu-Natal, Pietermaritzburg, South Africa; 8 Department of Genetics, Evolution and Environment, University College London, London, United Kingdom; Leiden University, NETHERLANDS

## Abstract

Global broiler production and consumption levels continue to rise. South Africa’s broiler system is dominated by commercial production and formal retail trade, with competition from cheap imports. Local broiler policies have narrow, production-driven, short-term aims for industry growth and national food security. However, these have unintended consequences that undermine the system’s future sustainability. Using a food systems approach, this study developed a qualitative system dynamics model of the South African commercial broiler system and used it to engage stakeholders in policy discussions within the boundaries of health, nutrition, and environmental sustainability. A problem statement and key system elements were drawn from a previously published qualitative study and were validated by 15 stakeholders via an online questionnaire. From this, a seed model was developed, expanded into a larger model, and shared in a modular format with stakeholders in virtual meetings, on an individual or institutional basis, for feedback and validation, and for discussion of areas for policy consideration. Refinements were incorporated into the modules, policy considerations were summarised, and crosscutting issues were identified. The model demonstrated the system’s complexity, interlinkages, feedbacks, reinforcing and balancing loops, and behaviour archetypes. The modular presentation format created a suitable platform for stakeholder engagement. Current policies focus on local commercial production, formal markets, and affordability without cognisance of the broader system represented by the model. Inequality pervades throughout the system. Commercial producers, linked to large supermarkets and fast-food chains, dominate the system, presenting barriers to entry. Affordability is unintentionally traded off against non-communicable disease risks through brining of most frozen products, and ultra-processing of fast-food items. Foodborne disease control is critical, given the proportion of vulnerable individuals, and greater coherence of food safety policy is urgently needed. The environmental footprint of broilers, whilst less than that of ruminants, deserves closer scrutiny based on its dependence on intensive cereal production for feed. This study’s food systems approach provides a system-wide perspective and a foundation for policymakers to develop more integrated and transformative policies.

## 1. Introduction

Less than a decade remains to achieve the Sustainable Development Goals (SDGs), and food systems require urgent change to contribute positively [1]. Currently, food systems fail to resolve the triple burden of malnutrition, they contribute to the burdens of diet-related non-communicable disease (NCD) and foodborne disease (FBD), and are environmentally unsustainable [1]. Livestock-derived foods (LDF), whilst providing a rich source of highly bioavailable nutrients and essential micronutrients [2], are specifically criticised as being unsustainable [3]. Countries undergoing rapid economic transition show the greatest increases in LDF consumption, commonly associated with changes in the food system and the food environment [4, 5]. Such rises in consumption levels in low and middle-income countries (LMICs) increase FBD risk due to a lag in food safety capacity development [6].

Globally, broiler chicken meat shows the greatest recent growth in production (doubling between 1998 and 2018), currently accounting for over 35% of the total meat production, second only to pork [5]. The United States (US), European Union (EU), China, and Brazil are the world’s top broiler producers, utilising large-scale and intensive production systems that are dependent on concentrate feeds [5, 7]. Broiler meat is presented as the healthy and least environmentally damaging meat option by the EAT-Lancet Commission on healthy diets from sustainable food systems [8]. However, broiler meat’s intensive production system’s reliance on the equally intensively produced cereals for its feed, contributes the most to its environmental impact [9]. Commercial broilers’ genetic advancements have concentrated on only a few breeds suited to intensive production, and on maximising feed conversion rates, and shortening maturation time, but this has not been without trade-offs. These include increased mortality rates, welfare concerns, and vulnerability to diseases like avian influenza [10, 11], and a reduction in the protein content and increase in fat content of the meat [12].

The LDF system in South Africa presents several SDG-related challenges to policymakers, which would be equally applicable in other LMICs. The country’s population has increased by over 30% since 2000, and meat consumption per capita (60kg/annum) is the continent’s highest [5, 13]. Surpassing the global consumption trend, broiler meat, which is the most affordable meat option, makes up 60% of meat consumption, with over 75% being produced by seven commercial producers [5, 14]. (Commercial producers in the South African context are regarded as large-scale, business-orientated producers on privately owned farms, using intensive production systems with high investment and inputs, and who are highly engaged in the formal market]). Alongside these high levels of meat consumption, stunting prevalence in under 5-year-olds is 22%, while adults classified overweight and obese exceed 31% in men and 68% in women [15, 16]. In addition, micronutrient deficiencies are high, with iron-deficiency anaemia prevalence, for example, at 25% [15, 16].

Currently, broilers are the largest contributor to the country’s agricultural production in terms of value and tonnage [17]. Over the last decade, favourable import tariffs allowed broiler meat imports to rise by almost 45% to peak at 0.57 million tons in 2018, compared to 1.6 million tons produced locally, which has shown little change over the previous 5 years [18, 19]. Only 11% of South Africa’s total land is suitable for cropping, and 3% is used for cereal production, most of which is rainfall dependent and vulnerable to climate change [20, 21]. Although South Africa is largely self-sufficient in maize, soya is imported to meet shortfalls in local production, and to fulfil the needs of the livestock feed industry, whose primary output is broiler feed [22]. From a food safety perspective, South Africa experienced the largest global foodborne listeriosis outbreak in 2017–18, which was linked to a low-cost processed meat product that contained mechanically deboned meat (MDM), including that of broiler origin [23, 24]. Food safety is particularly important in South Africa, given the proportion of the population at greater risk, including under 5-year-olds, and those living in poverty, with malnutrition, or HIV/AIDS [25, 26].

The South African food policy environment has been criticised for lacking coordination and coherence, with 15 government departments responsible for various aspects [27]. Their food system policies are characterised by centralised decision-making, a lack of understanding of the challenges’ complexities, and a failure to recognise the policy and governance benefits of wider non-government stakeholder engagement [28, 29]. Agricultural development policies, including those associated with broiler production, have pursued the global model of intensive, commercial systems to maximise outputs, efficiencies and profitability, aimed at national food security goals, without the inclusion of social, cultural, and ecological aspects, or recognition of the links to health and nutrition [30, 31]. The country’s food security focus is regarded as being rooted in capitalist development models that miss the broader socio-political and economic change required for genuine food system transformation [32]. Similarly, nutrition research and related policy development, lack evidence for the links between consumer nutrition outcomes and changes in their food environment (i.e. the availability, accessibility, affordability, desirability, convenience, marketing, and properties of food sources and products) [33, 34]. South Africans increasingly buy more, and grow less, of the food they consume, and the impacts of these shifts in the food environment on consumers’ choices are yet to be identified [35]. Formal food retail, being closely linked to commercial production in South Africa, shows similar development, and follows a global North trajectory. The top seven food retail companies distribute 80% of South Africa’s food [36], and their expansion into rural areas has outcompeted traditional independent retailers and rural markets [37, 38]. Similarly, the country’s fast-food industry is burgeoning in both urban and rural locations [39]. Outlets are visited at least once a month by most consumers, and broiler meat, following global trends, plays a dominant role in the products on offer [39–41].

The national and global challenges facing food policymakers are typically complex and interlinked, and defined as wicked [42]. Wicked problems have ill-defined boundaries, no definitive solution, and are characterised by stakeholder disagreement, resistance to evidence, research, and reasoned debate [43]. Intervention attempts are also prone to delivering unintended consequences [42, 43]. The main barriers within food system policymaking relate to stakeholders, often the most powerful, having short-term strategies, siloed agendas, misaligned incentives, pulling in different directions, and being motivated by factors other than those of health and sustainability [1]. Established approaches to policy, typified by linear, sectoral thinking, and a reliance on quantitative data, may merit highly scientific and technically natured problems, however, wicked problems require more integrated, systems approaches [44, 45].

A food systems approach, or “food systems thinking”, is the paradigm shift championed to transform food-related policymaking, and to manage the complex and wicked problems associated with food [46–48]. Systems thinking focuses on understanding the system’s structure, boundary setting, identifying key elements and their interrelationships, causal links, and feedback loops [49]. It helps shift policymakers’ attention from isolated issues towards understanding the interlinkages of drivers and potential unintended consequences [47]. In a food systems approach, systems thinking is applied to the food system [46]. The latter is defined as the activities, outcomes, and actors involved in the “farm-to-fork” process, and the associated economic, social, political, environmental, and health drivers [50]. System dynamics (SD) modelling offers tools that complement systems thinking [51], and that can operationalise food systems approaches, and be used to inform policy [52, 53]. SD modelling is highly iterative and often involves qualitative and quantitative elements [54]. Apart from the quantitative aspects of formulation and simulation, SD modelling facilitates problem-solving, particularly when stakeholders are engaged [55, 56]. Qualitative models can be used as a standalone tool for policy guidance in systems that have broad boundaries and involve multiple stakeholders, and where quantification is difficult, hampered by uncertainty, or restrained by time and financial resources [57, 58]. Several examples of these models relate to food systems, agriculture, and food security [59–62].

Given the wicked problems and complexity of food systems in South Africa, and in countries undergoing similarly rapid development and transformation, there is a need to develop policymaking processes, based on a system-wide perspective, which are more suited to dealing with the complexity of such challenges. This research forms part of the Sustainable and Healthy Food Systems (SHEFS) programme, which aims to provide policymakers with novel, interdisciplinary evidence to define future food system policies that deliver nutritious and healthy foods, in an environmentally sustainable, and socially equitable manner. This study aimed to develop a qualitative SD model of the commercial broiler food system in South Africa, and to use it to engage with stakeholders to identify key policy areas within the boundaries of nutrition, health, and environmental sustainability. The South African broiler system is currently being directed by policies based on the Poultry Master Plan (PMP), which was agreed at the end of 2019, and set to run for three years [63]. It is a partnership between government and industry stakeholders, focussing on the growth and development of local industry and the associated feed value chain. Support for local production is planned through imposing higher tariffs on meat imports and seeking to improve export opportunities, both regionally and in the EU. Partnerships and investments are planned to support local maize and soy production to reduce the need for imported ingredients. In return, the industry is expected to facilitate a greater degree of participation, employment, and ownership by Black actors throughout the value chain. Given the prominence of the PMP policies in the future of the broiler system, the results from this research aim to help position the PMP within the wider broiler food system in South Africa, and provide insights for future policies. In addition, several insights may be transferable to the South African LDF system, and to comparable intensive broiler systems elsewhere.

## 2. Methods

**Fig 1:**
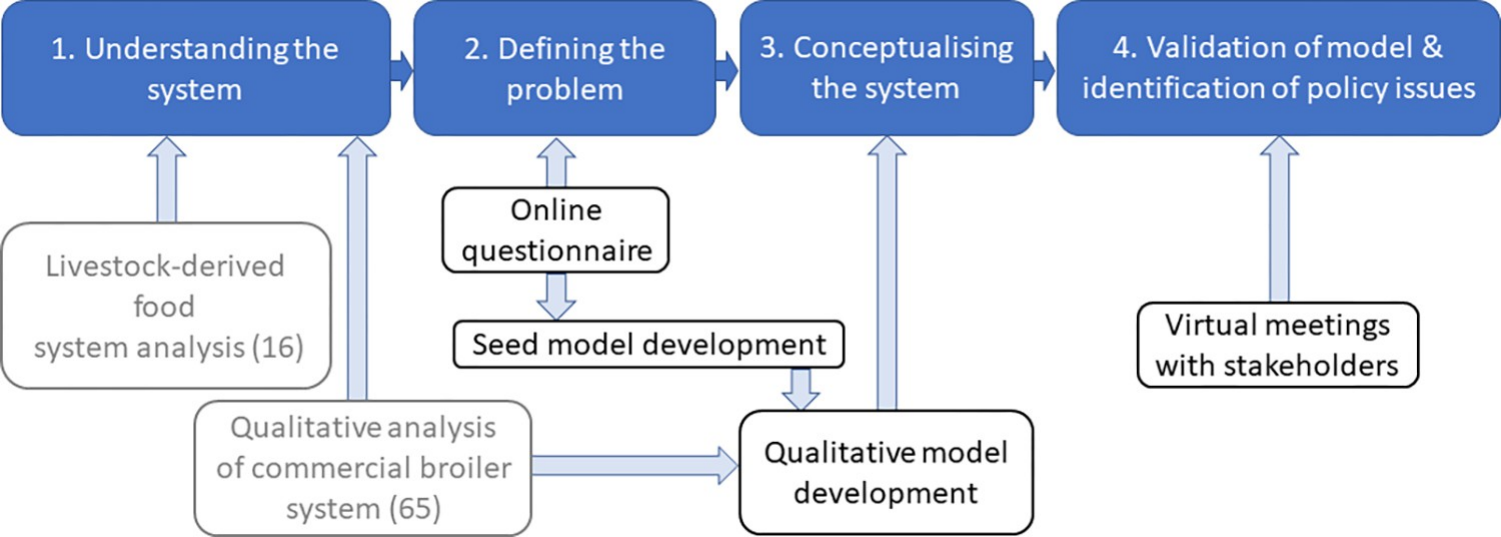
Four stages of research (in blue) and the inputs for each (previously published in grey, current study in black).

A food systems approach was used to develop a qualitative SD model, using systems thinking and tools from SD modelling, and stakeholder engagement. Model development followed the qualitative steps described by Martinez-Moyano and Richardson [54], namely, understanding the system, defining the problem, and system conceptualisation. An outline of the stages of model development, validation, and analysis, and the inputs that were used in this study is presented in Fig 1.

### 2.1 Understanding the system

An understanding of the broader LDF system had been gained through preceding research that included a LDF systems mapping workshop with stakeholders, and the building a conceptual SD model of the LDF system [64]. This preceding work highlighted the significance of the commercial broiler system within South Africa’s wider LDF system, leading to it being the focus of further research within the SHEFS programme. To understand the commercial broiler system, a qualitative study of the commercial broiler system was conducted and the results are published elsewhere [65]. A concurrent study focused on the challenges facing small-scale and “emerging” producers [66]; the latter term describes those transitioning from small-scale to medium- and large-scale commercial farming [64]. The qualitative commercial broiler system study was based on semi-structured interviews conducted with stakeholders in May-June 2019. Stakeholders were selected purposively from those identified from the preceding LDF system research, online searches, professional networks, and snowballing. We sought representation throughout the value chain, and within the project boundaries. Invites were sent by email and included background information on the project. Twenty-nine stakeholders (13F, 16M) agreed to take part, and they included representatives of large, medium, and small-scale commercial producers, importers, input providers (feeds and medicines), animal health service suppliers (public and private veterinary practitioners), human health laboratory specialists, academics and researchers in natural resources, economics, animal health, human health, and nutrition, and representatives from the broiler producer association, non-governmental organisations, and government departments and agencies. Interview transcripts underwent qualitative analysis, with literature searches to triangulate findings.

### 2.2 Defining the problem

Based on this system-wide understanding, a problem statement was developed. It was kept broad to encourage stakeholder ownership and engagement [67], and was framed within the research boundaries for this study to infer stakeholder agreement on these [68]. We used the script from Handel and Kleemann [67] as a guide for an online questionnaire to agree on a problem statement and facilitate identification of the related key elements. The original group of 29 interviewed stakeholders were invited via email in September 2019 to participate in a follow-up online questionnaire using Google Forms (S1 Text). The questionnaire presented a problem statement and a list of 11 system elements, based on a preliminary analysis of the interviews and relevant literature searches. In the questionnaire, participants were asked if they agreed with the statement or not, and if not, to offer refinements. They were also asked to identify the main elements that they related to the problem, from the list of 11 provided, with the option to add others if necessary.

### 2.3 Conceptualising the system

System conceptualisation is the process of generating a visual representation of the system, and the dynamic hypothesis of the cause of the problem [63]. The questionnaire results were used to develop a seed model, which aims to provide a basic representation of the problem situation [51]. Individual elements were disaggregated, and interlinkages and feedback loops between them were illustrated. The seed model was later used to share findings and to introduce the basics of SD modelling in follow-up stakeholder meetings, but also formed the foundation of the qualitative SD model, both of which were created using software by Vensim PLE for Windows, Version 8.1.1 [69].

Building the qualitative model began with disaggregation of individual seed model elements. The use of qualitative data, based on individuals’ mental models, is the main source of information in SD model building, particularly when there are numerous soft variables involved [70]. Interview data, literature, and existing knowledge are used to conceptualise the system [51]. Hence, using the findings from our previous published studies, we developed the model based on our knowledge of the wider LDF system [64] and the qualitative analysis of the broiler stakeholder interviews, and the related literature searches [65]. In addition, we used the logical reasoning and experience of the interdisciplinary team of co-authors and fellow researchers within the SHEFS programme, to add further elements, causal links, conceptual stocks and flows, reinforcing and balancing feedback loops, and system archetypes. A reinforcing loop is one where an increase in a variable, when traced around the loop, leads to a further increase in itself, while a balancing loop is one where an increase in a variable leads to a counterbalancing decrease in itself. System archetypes classify generic patterns of behaviour over time (in particular counterintuitive behaviours), and demonstrate intended and unintended reactions and delayed reactions [71]. They are recognised as powerful tool to understand and communicate the underlying system’s dynamic behaviour [71]. These were identified based on descriptions provided in the literature [71, 72], and on the research team’s previous experience. The model was kept within the boundaries of human health, nutrition, and environmental sustainability. Whilst noting that the whole model demonstrated the system’s complexity, for ease of presentation to stakeholders it was split into four modules, based broadly on the boundary categories and the model’s logical layout. For each module, a narrative was developed for explanation to stakeholders in subsequent meetings.

### 2.4 Model validation and identification of policy issues

The modules and narratives were used as a tool to engage with stakeholders and identify key areas of policy within the boundaries of nutrition, health, and environmental sustainability. A planned return visit to South Africa for face-to-face meetings with stakeholders was not possible due to the COVID-19 pandemic. As an alternative, the original 29 interviewees were invited to participate in individual virtual meetings in December 2020. A further 16 stakeholders who were unable to take part in the first interviews, or had been subsequently identified through vertical and horizontal networking, were also invited. The 35 email invitations included background information and a link to a 10-minute recorded video presentation. The latter’s purpose was to share the key findings from the interview and questionnaire analysis, to introduce the basics of reading a qualitative SD model, and to present the seed model and its narrative. The aim of the meetings was to share detailed findings of the research via the model, to have stakeholders identify gaps, suggest corrections and to validate the model, and, finally, to identify key policy areas for consideration that would enhance outcomes, and mitigate unintended consequences. A total of 15 individuals from nine institutions took part (eight from the original group of 29, and the remainder were new). They included representatives from the broiler industry, the retail and manufacturing industry (including a nutritionist), a not-for-profit economic research institute, an importer, a veterinary and animal health service provider, an animal feed company, a natural resource researcher, broiler production researchers, and an antimicrobial resistance researcher. Written consent to participate and for video recording of the meeting was acquired using a consent form that was provided and returned by email. Ethical approval for this research was gained from the Royal Veterinary College’s (University of London) Social Science Ethical Review Board (URN SR2018-16240).

Meetings were conducted on an individual or institutional basis, via Zoom video call, with up to three co-authors facilitating. Following an introduction and project outline, the whole model was presented for visualisation, followed by the four individual modules. For each module a narrative was presented whilst progressively revealing small groups of elements and associated links until the full module was visible. Finally, suggested issues for policy consideration were highlighted on the module. These had been drawn from the previously conducted broiler stakeholder interviews and their qualitative analysis [65], and those identified by the authors through identification of nexus points within the model. After each module presentation, participants were invited to feedback on any areas requiring expansion, to identify any major omissions or misinterpretations, and to suggest edits or additions to the highlighted policy issues, including barriers or opportunities. Meeting recordings were transcribed and used to review and summarise interviewees feedback and comments. Policy issues raised in the meetings were then analysed by the authors against policies central to the PMP, to identify the degree of coverage by the latter and to identify gaps. This was done for each module, but crosscutting policy issues were also included separately. The results for each of these analyses were tabulated separately. The stakeholders broadly responsible to respond to each policy issue, and those likely to benefit from it, were also identified and included in the tables.

## 3. Results

### 3.1 Online questionnaire, problem statement and seed model

**Fig 2:**
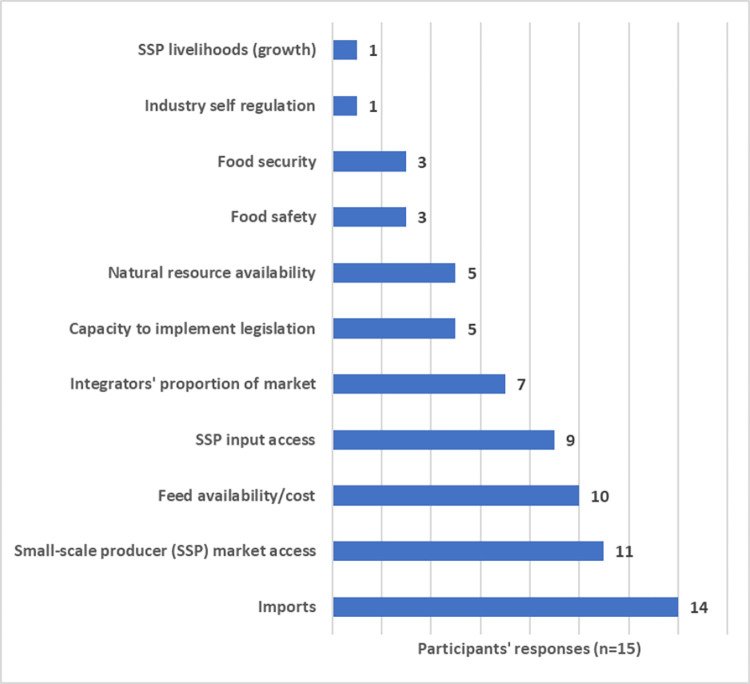
Number of participants selecting proposed elements they considered linked to the problem statement. (SSP = Small-scale producers).

Of the 29 original interviewees, 15 responded to the questionnaire. The following problem statement was proposed: “The current broiler system in South Africa is under strain to produce sufficient food in an environmentally sustainable way, that is safe and nutritious, and meets the needs of a growing population in a socially equitable manner.” All participants agreed, apart from one who responded that the strain was caused by imports. The list of 11 elements, drawn from the initial broiler stakeholder interviews, and the number of participants that identified each as relevant to the problem statement, is presented in Fig 2. These elements were used to represent the main elements behind the problem statement and to develop the seed or foundational model as a first stage in the SD model building (S2 Text).

### 3.2 Qualitative SD model and individual modules

The seed model, together with the previously conducted literature searches and qualitative analysis of interviews, formed the basis of the qualitative SD model development (S1 Fig). The full model was separated into four modules, each described in the sections below. All model and module figures follow standard labelling protocols for SD modelling [51]. Polarity of arrows indicates direction of change in the variable to which the arrow points, when a change occurs in the variable preceding the arrow (+ indicates change in the same direction,—indicates change in the opposite direction). Reinforcing and balancing loops are indicated with Rn and Bn respectively. Conceptual stocks are in rectangles, with flows in and out of stocks indicated by thick, linear arrows with hourglass shaped “valves”. Within each individual module, variables that are present in (and linked to) other modules are colour coded in blue. Each module has been modified from those originally presented to include additional variables suggested by stakeholders during meetings (in brown). Policy considerations that were presented to, or suggested by, stakeholders within each module are summarised (and positioned against the PMP coverage) in a table at the end of each section. Those responsible for the area of policy and those who benefit from it are also listed in each table. Cross-cutting policies identified by authors during the analysis are presented in Section 3.2.5.

#### 3.2.1 Production and imports

**Fig 3:**
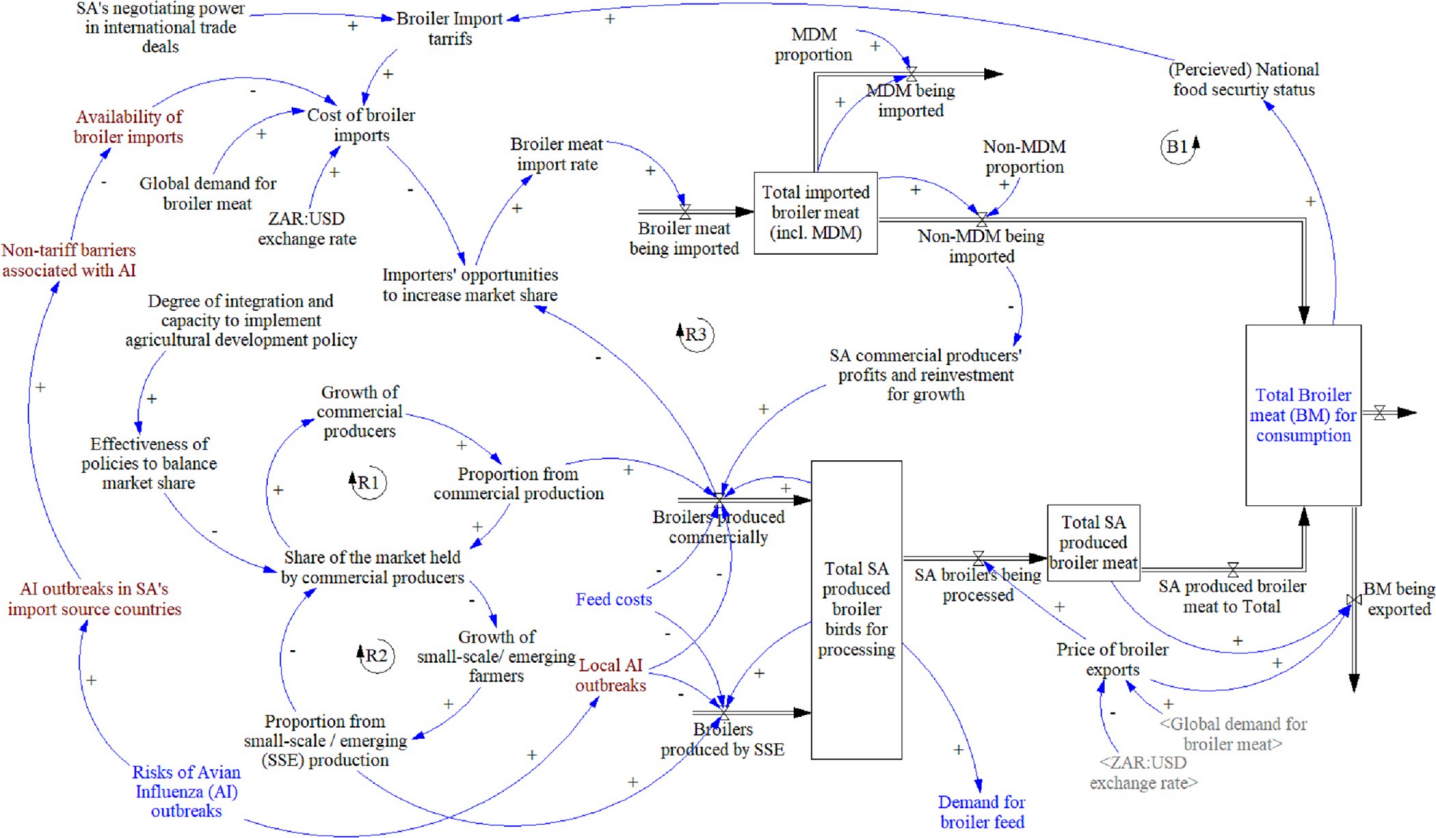
Broiler production and imports module. Key: Blue text: elements linking to other modules, Brown text: elements added after meeting with stakeholders, Grey text between < > are duplicated variables within the module. Abbreviations: AI = Avian Influenza, MDM = mechanically deboned meat, SA = South Africa(n), SSE = Small-scale and emerging, USD = United States Dollar, ZAR = South African Rand.

This module (Fig 3) shows the dominance of commercial producers in reinforcing loops (R1 and R2), which form a “success to the successful” archetype [71]. Five vertically-integrated, commercial producers hold >75% of the market [17], which reinforces their growth and their production outputs, building their advantage over small-scale and emerging producers, through access to inputs, economies of scale, production efficiency, quality assurance, and policy influence. Despite the creation of post-apartheid national and agricultural development policies, they have been insufficient to shift this archetype due to a lack of integration and implementation capacity. Production from both commercial and small-scale and emerging farmers are affected by feed costs, and the total birds produced locally determines the demand for broiler feed (see Section 3.2.3). A small proportion (3%) of locally produced broiler meat is exported [14], which is dependent on local production stocks, and the price of exports, which in turn is impacted by global demand and the currency exchange rate.

Imported broiler meat has two main components namely MDM (33%), which goes into processed meat products, and non-MDM [14]. The latter is primarily frozen bone-in broiler pieces (45% of total) and the remainder consists of clean offal, whole carcases, and other categories [14]. Non-MDM products compete directly with those produced locally. Both the locally produced meat and imported non-MDM meat contribute to the total broiler meat available for distribution and consumption (see Section 3.2.2). This total influences the perceived national food security status, which in turn influences the government’s import tariff setting policies, although these may ultimately depend more on other international trade deals. Government policies to lower import tariffs, in an attempt to bolster food security, is a “fix that fails” archetype [71], where this short-term fix has an unintended consequence of undermining the commercial producers’ contribution to food security, thereby failing in its aim. Import tariffs, together with the exchange rate and the global demand for broiler meat, contribute to the overall cost of broiler imports. The cost of imports impacts on importers’ opportunities to increase their market share and increase the rate of imports. This forms the start of the “fix that fails” archetype, with a reinforcing loop (R3) that demonstrates that if importers’ market share increases, imports increase, outcompeting local broiler meat, diminishing local profits and growth (of primarily commercial producers), which creates more opportunities for importers. The same reinforcing loop can work in favour of commercial producers, i.e., if their production increases, they will reduce importer opportunities, decrease imports and improve their own profits for growth and reinvestment. Balancing loop (B1) indicates that if either imports or local production increase, it will improve perceived food security, and potentially allow government to impose stricter imports, reducing imports until such time as the total meat available reduces again.

Stakeholders interviewed generally agreed that the module and narrative was an accurate representation of their understanding of the system. A suggested addition was the impact of avian influenza outbreaks on international trade and on local production.

Policy areas presented for consideration were generally agreed upon by individual participants, but further specifics were suggested, and most of these were considered within the PMP’s vision (Table 1).

**Table 1:** Summary of production and imports policy areas for consideration. Original ideas presented in meetings are in plain font, and stakeholder additions in italics (PMP = Poultry Master Plan).

Production and Imports			
Policy areas for consideration	PMP coverage	Responsibility	Benefiter
Address drivers of the dualistic production system, remove barriers to participation, and support small- and medium-scale broiler producers	Partial	Government and local industry	Small- and medium-scale broiler producers
Government support to balance local vs. imported meat (within context of food security, and complexity of trade deals), through tariff adjustments on bone-in meat imports	Yes	Government	Local industry
*Relaxation of non-tariff barriers (relating to avian influenza) to allow more competitive access to import markets*	No	Government	Importers
*Stricter control of illegal imports* (and the capacity to implement regulations)	Yes	Government	Local broiler producers, consumers
Opening export opportunities *(by addressing barriers to meet high standards*, *and limitations in capacity to monitor*, *certify and provide assurances)*. *Alternatively*, *prioritising more realistically achievable export opportunities*.	Yes	Government and local industry	Local broiler producers
*Supporting retailers to “Buy Local” (with agreed quota) to support neighbouring small-scale producers*	Yes	Government and local retailers’ association	Local small- and medium-scale broiler producers and retailers

#### 3.2.2 Distribution and consumption

**Fig 4:**
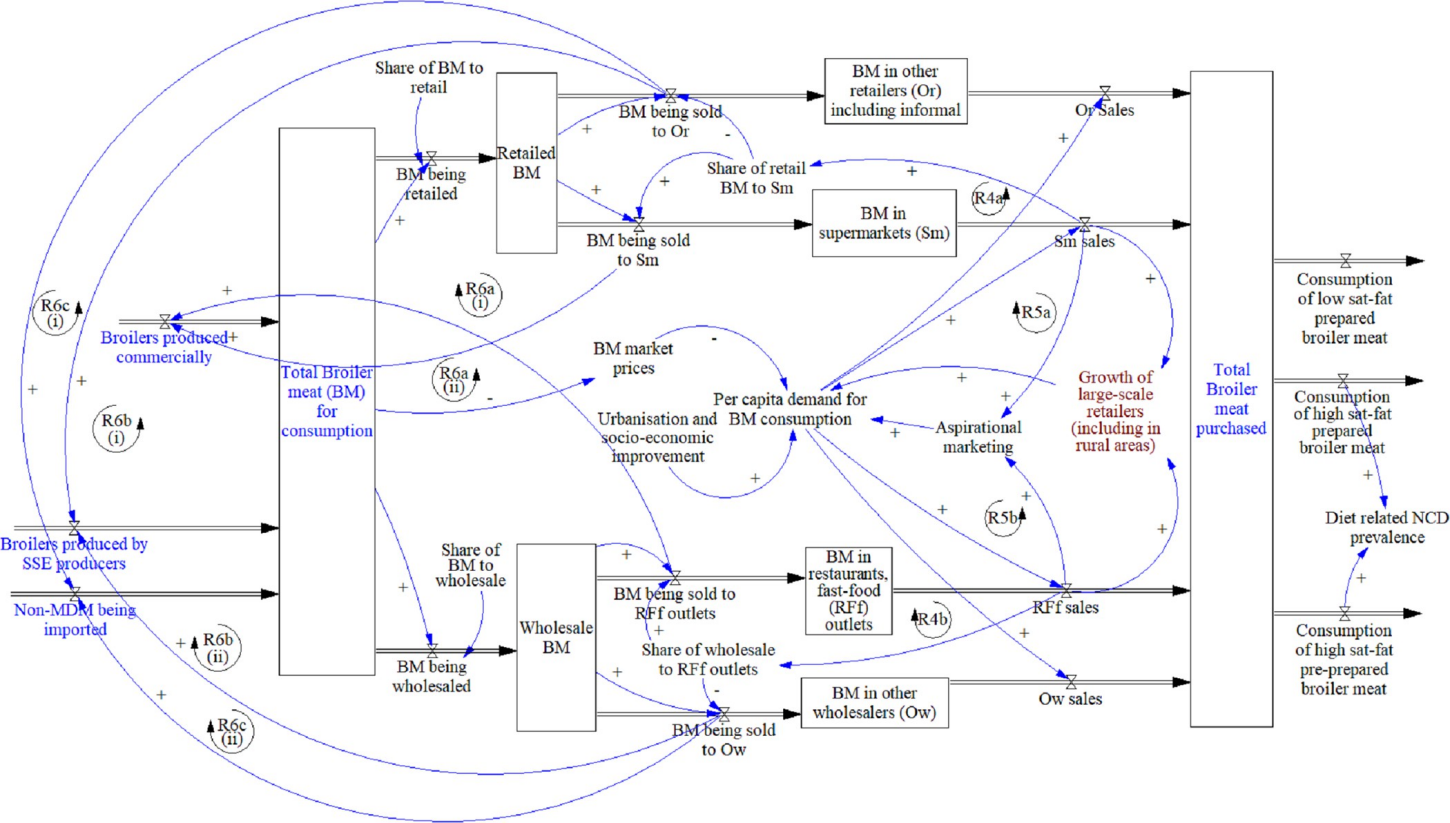
Distribution and consumption module. Key: Blue text: elements linking to other modules, Brown text: elements added after meeting with stakeholders. Abbreviations: BM = broiler meat, SM = supermarkets, Or = other retailers, RFf = restaurants and fast-food, Ow = other wholesalers, NCD = non-communicable diseases.

This module (Fig 4), links back to the previous one via the total broiler meat for consumption variable, and it demonstrates the retail and wholesale flows of product. Both have a dominant flow, namely, supermarkets for retail, and restaurants and fast-food outlets for wholesale, and the balance of each flows through other retailers and wholesalers, including less formal enterprises. Supermarkets dominate with an estimated 80% of food retail passing through the top seven companies, and, whilst the fast-food sector has many small and informal actors, the bulk is represented by large, local and international franchise companies [36]. These market shares are supported by reinforcing loops (R4a and R4b), where sales drive the proportion distributed through these flows. Sales from all outlets are driven by per capita demand, which, in turn, is affected largely by prices, and also strongly influenced by the volumes passing through large supermarkets and fast-food outlets, who invest in aspirational marketing to advertise their range of products and prices (R5a and R5b). Equally, drivers of demand include urbanisation and socio-economic improvement. Distribution through formal channels (supermarkets and restaurants/ fast-food outlets) feeds back primarily to commercial production (R6a(i)/(ii)) due to their demand for greater traceability, quality, and safety standards. By contrast, distribution through other retailers and wholesalers, and informal outlets, feeds back primarily to small-scale and emerging producers (R6b(i)/(ii)), and import suppliers (R6c(i)/(ii)), as they as less demanding of the same standards as formal distributors. These reinforcing loops are ultimately controlled by the dominant market share loops of R4a and R4b, and are underpinned by a “success to the successful” archetype that increases the market share of the formal system, whilst undermining the share of others. At the consumer level, purchased broiler meat can be consumed in a variety of ways, dependent on in-home or pre-purchased cooking methods, with nutritional impacts varying accordingly.

Individual participants generally agreed that the module and narrative was an accurate representation of their understanding of the system, however some additions were suggested. A gap identified was the unpacking of the impact of local brining on prices compared to imports. However, whilst noted, this would require extensive disaggregation of the “Total broiler meat for consumption” variable into various products, which was considered beyond the scope of this model. The pervasion of fast-food outlets in even rural food environments was also suggested for inclusion.

Policy areas presented for consideration were generally agreed upon, but with some further contributions from participants (Table 2).

**Table 2:** Summary of distribution and consumption policy areas for consideration. Original ideas presented in meetings are in plain font, and stakeholder additions in italics (PMP = Poultry Master Plan).

Distribution and Consumption			
Policy areas for consideration	PMP coverage	Responsibility	Benefiter
Address drivers of dualistic distribution system (extension of those addressing dualistic production system), remove access barriers and support development of small- and medium-scale enterprises	Partial	Government and local retailers’ association	Small- and medium-scale retailers
Consider drivers of increasing demand (price, supply, marketing) and related nutritional outcomes of increased consumption (nutrition security vs. obesity and related NCD). *Affordability unpins nutritional security in low-income groups*, *and equally overconsumption in those with more disposable incomes*. *Lower cost options products (e*.*g*. *soup packs*, *containing trimmings from expensive cuts) may have little nutritional worth and negative nutritional outcomes*.	No	Retailers, importers, and broiler producers	Consumers

#### 3.2.3 Feed and environment

**Fig 5:**
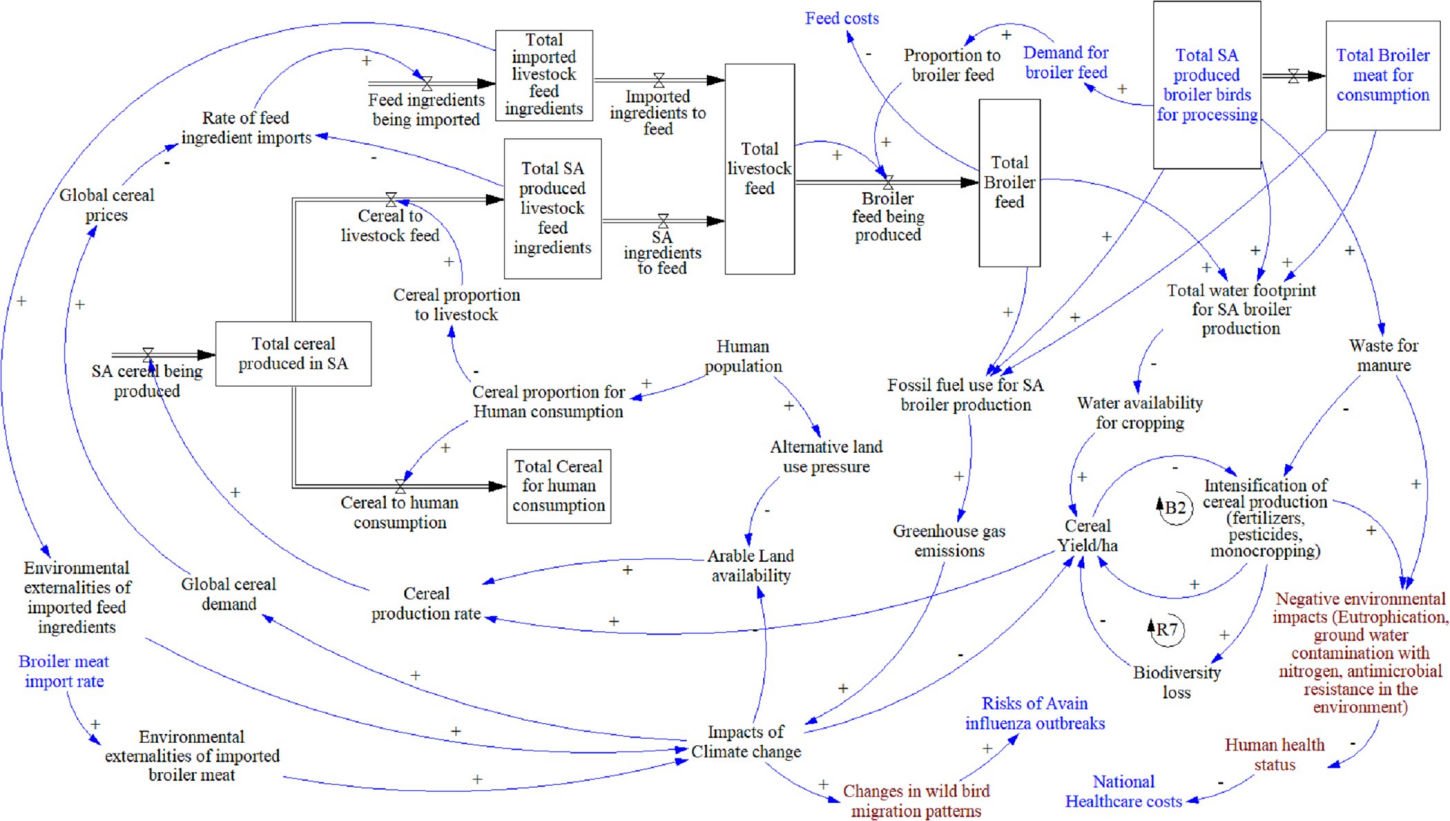
Feed and environment module. Key: Blue text: elements linking to other modules, Brown text: elements added after meeting with stakeholders.

This module (Fig 5) links to the production and imports module, given that commercial broiler production is totally dependent on cereal-based concentrate feed. The cereal production rate in South Africa is affected by the yield per hectare and the arable land availability. The latter is threatened by alternative land use pressure, which ultimately is linked to human population expansion. The yield per hectare is driven through intensification of production, theoretically within a balancing loop (B2), if demand remained the same. Intensification, through the use of pesticides, herbicides, and inorganic fertilisers, is a “fix that fails” archetype, since it reduces biodiversity and related ecosystem services, which in turn reduces yields, and drives the need for further intensification in a reinforcing loop (R7).

Cereal production is divided into that for human, and that for livestock consumption, the proportion being driven primarily by the human population. Livestock feed ingredients are primarily locally sourced, with imported ingredients meeting any deficits, which are mostly in terms of soy. Imports are affected by global prices and global demand. The proportion of total feed going to broilers is driven by demand, based on the production levels (as per Production and Imports module). The total broiler feed produced will influence feed costs, which also links back to the same module.

On the environmental impact side, feed and broiler production create a combined water footprint and a combined fossil fuel use, and broiler production also leads to excrement waste. The latter, when used as an organic fertiliser, arguably reduces the need for inorganic fertiliser, whilst the water footprint reduces the water available for crop use and reduces cereal yields. Fossil fuel use leads to greenhouse gas emissions, which add to the impacts of climate change, which, in turn, directly affect yields, and indirectly affect production, as more arable land becomes marginal due to increasing aridity. Climate change is also a product of the environmental externalities of imported feed ingredients and imported broiler meat.

Individual stakeholders generally agreed that the module and narrative was an accurate representation of their understanding of the system. A suggested addition was that water and fossil fuel usage should include the processing and distribution phases, and that links demonstrating waste’s environmental impacts (nitrogen, eutrophication, and antimicrobial resistance) were needed. Climate change’s impact on wild bird migration patterns and risks of avian influenza, both internationally and locally, was also suggested for inclusion.

Policy areas presented for consideration were generally agreed upon with some refinements added by participants (Table 3). Much of these were deemed outside the scope of the PMP.

**Table 3:** Summary of feed and environment policy areas for consideration. Original ideas presented in meetings are in plain font, and stakeholder additions in italics (PMP = Poultry Master Plan).

Feed and Environment			
Policy areas for consideration	PMP coverage	Responsibility	Benefiter
Support local cereal production to manage dependence on imports to meet feed ingredient shortfalls, primarily soy. *Remove soy import tariffs to reduce feed costs but keep local production competitive through investment and support*.	Yes	Government and local soy producers	Local soy producers, feed manufacturers, broiler producers
*Develop human soy value chain*, *to benefit broiler feed industry with local oilcake by-product*, *whilst providing low-cost plant-based protein foodstuff*.	No	Local soy value chain actors	Feed manufacturers, broiler producers, consumers
Reconsider international pricing structure of ingredients, to lower feed costs locally.	No	Government	Feed manufacturers and broiler producers
Balance land-use and cereal production for human food and livestock feed.	No	Government (incentives), local cereal farmers	Consumers, environment
Improve resilience to climate change impacts of the broiler system’s natural resource dependence	No	Local broiler and cereal producers	Broiler and feed producers
Mitigate environmental impact and improve environmental sustainability throughout broiler system	No	Government, cereal producers, local broiler producers, and processors	Environment

#### 3.2.4 Food safety and foodborne disease surveillance

**Fig 6:**
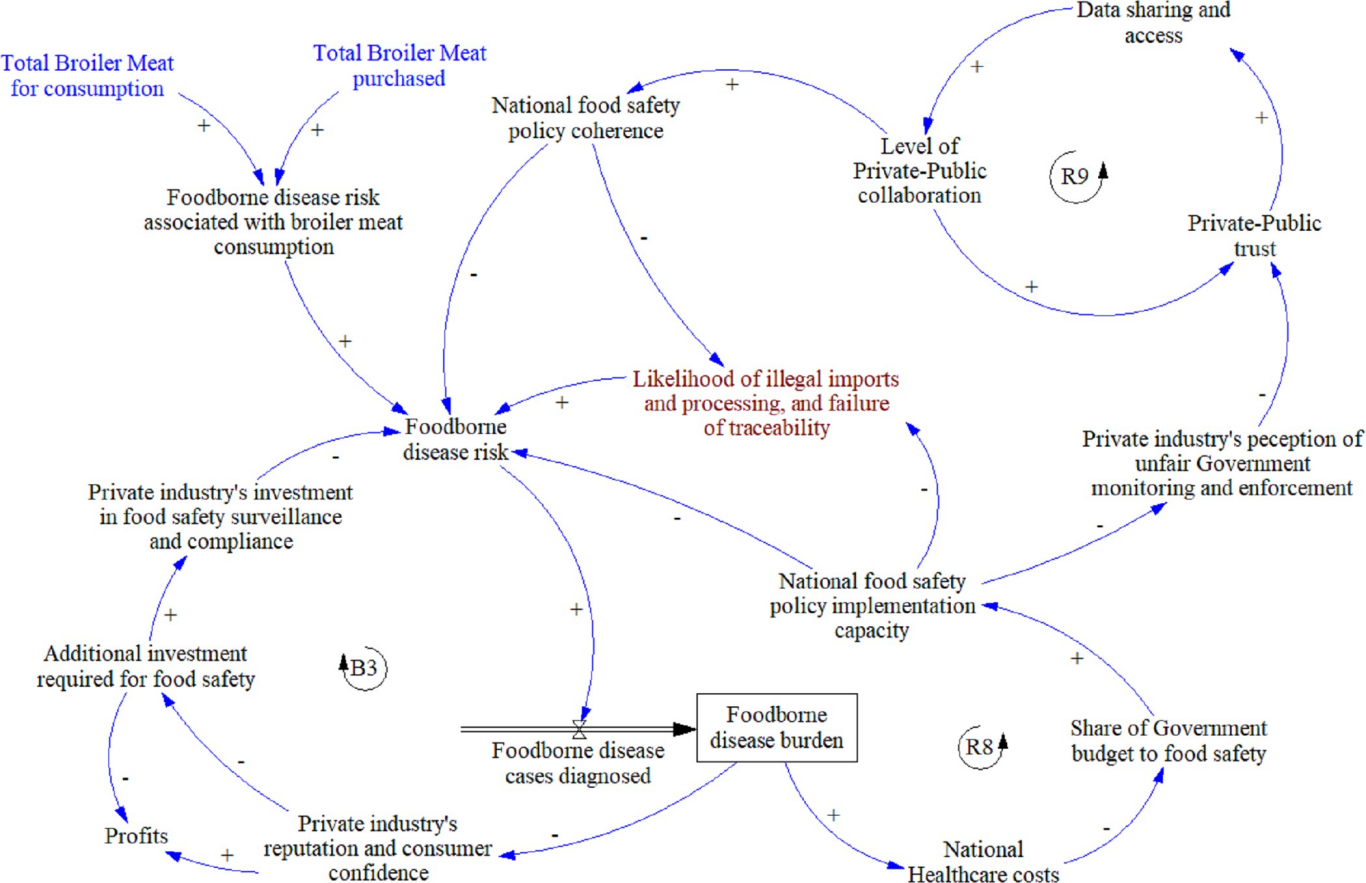
Food safety and foodborne disease surveillance module. Key: Blue text: elements linking to other modules, Brown text: elements added after meeting with stakeholders.

This module (Fig 6) links back to the Distribution and Consumption module via the total broiler meat purchased. Overall FBD risk and the associated FBD burden has a balancing loop (B3) through the private sector, where the threat of the industry’s reputation and consumer confidence being undermined, drives further investment in food safety surveillance and compliance with regulations, thereby reducing FBD risk. In contrast, the FBD burden increases national healthcare costs, eroding the government’s budget for food safety implementation, thereby increasing risk of FBD in a reinforcing loop (R8). When government’s capacity to implement food safety regulations is poor, the large players within private industry perceive that they are targeted unfairly with monitoring and enforcement, which undermines private-public trust. The lack of trust reduces data sharing and access, and further undermines collaboration and trust in a reinforcing loop (R9).

Individual stakeholders generally agreed that the module and narrative was an accurate representation of their understanding of the system. A suggested addition was the role of illegal activities in FBD risks (including illegal imports and illegal processing, i.e., thawing and refreezing), and the link between food safety and traceability of imported products.

Policy areas presented for consideration were generally agreed upon, with some refinements and additions (Table 4). Most of these were deemed outside of the PMP’s scope.

**Table 4:** Summary of food Safety and foodborne disease surveillance policy areas for consideration. Original ideas presented in meetings are in plain font, and stakeholder additions in italics (PMP = Poultry Master Plan).

Food Safety and Foodborne Disease Surveillance			
Policy areas for consideration	PMP coverage	Responsibility	Benefiter
Improving related policy coherence and implementation *(to ensure a balanced and consistent roll out)*	No	Government	Industry and consumers
Develop public-private collaboration based on improved trust	Partial	Government and local industry	Government, local industry, and consumers
Build on the shared interest in food safety, given proportion of population considered more vulnerable	No	Government and local industry	Government, local industry, and consumers
*Restrict imports to ready for sale packaged items labelled with country of origin*, *to reduce handling and repackaging*, *and improve traceability*.	Partial	Government and importers	Consumers
*Establish risk-based regulatory framework*, *supported by education and knowledge campaigns aimed at small-scale and informal providers*	No	Government and local industry	Government, local industry, and consumers

#### 3.2.5 Crosscutting policy considerations

Crosscutting themes (Table 5) for policy consideration were identified by authors in the process of collating results. These included the need for a paradigm shift from the narrow food security focus within the broiler system to a broader food system lens, addressing the inequality at the level of actors within the system and at the consumer level, and reforming of the food policy environment to improve policy coherence.

**Table 5:** Crosscutting themes for policy consideration (PMP = Poultry Master Plan).

Crosscutting Themes			
Policy areas for consideration	PMP coverage	Responsibility	Benefiter
Widening the narrow food security focus to the whole food system	No	Government, local industry, and importers	Government, local industry, and consumers
Inequality throughout system: Success to the successful. Big players dominate, from inputs and production, through to urban and rural food environments	Partial	Government, local industry, and importers	Local industry, and consumers
Inequality at consumer level: affordability relates to less healthy (nutrition and food safety) options	No	Government, local industry, and importers	Consumers
Food policy environment reform: with greater inclusion but consolidation/unification at level of decision making and implementation.	Partial	Government, local industry, and importers	Consumers, local industry, and importers.

## 4. Discussion

This study aimed to develop and use a qualitative SD model as a tool to engage with stakeholders and identify key areas of policy, within the boundaries of human health, nutrition, and environmental sustainability. Using a food system approach, this research positions the current broiler policies within the PMP, against the policy considerations identified by stakeholders, when examining the SD model of the wider broiler system.

The current PMP focuses on development of the local commercial industry, increasing participation of emerging farmers, and creating jobs throughout the value chain, whilst ensuring food security and increasing per capita broiler meat consumption. Previously, government policies attempted to address food security by relaxing import tariffs on broiler meat, to increase the supply and affordability for consumers [14, 73]. However, our analysis identified this as a “fix that fails”, one which undermines commercial production, and fails to bolster food security. This intervention also has the potential to develop into a “shifting the burden” archetype, where a dependency on the short-term fix develops [71]. This trend has been mirrored elsewhere in Africa, specifically Angola, where uncontrolled imports undermined the local industry creating an 85% dependency on imports [74]. Although the PMP’s current vision moves, instead, to support local production through the imposition of higher import tariffs, it retains a national food security focus.

Within the food security aim, the consequences of increasing average per capita consumption of broiler meat needs further unpacking. The Distribution and Consumption module identified the South African food environment as being dominated by large-scale actors. These include modern supermarkets, restaurants, and fast-food outlets, most of which belong to national and international chains. In addition, these are typically positioned in prime locations within shopping malls, the latter being pervasive even in rural areas. Such food environments increase processed food access and consumption, and recent evidence indicates their link to the country’s rising obesity prevalence [40]. As a result of competitive pressure from increased imports, large-scale integrated producers shifted their focus from supplying individually quick frozen (IQF) pieces to supermarkets, to developing preferential contracts to supply fast-food outlets that provided better returns. Whilst the PMP policies aim to ease import pressure, there is no guarantee that integrators will return to supplying IQF pieces. There is perhaps a greater likelihood of them strengthening contracts within the more lucrative and growing fast-food chicken market, supplying more unhealthy options, and leaving a shortfall in the stock of affordable IQF pieces.

The IQF pieces market is supplied by both commercial producers and importers. The bulk of broiler meat purchased from supermarkets is in the form of IQF pieces, and, due to the prevalence of supermarkets, it is the most commonly consumed product. The IQF pieces are also considered an important contributor to the affordability of broiler meat, which is in part due to the brining process. As a result of competitive pressure from cheap imports, brining was used as a coping mechanism for the local industry, to retain profitability [75]. The drive to increase per capita broiler consumption should consider the impact of the associated increased salt intake, via the consumption of brined IQF pieces, on hypertension related NCD. Given the PMP’s strategy to support local production through the imposition of higher import tariffs, government should consider steps to legislate for further reduction in brining, or an outright prohibition, as is the case in Brazil and Zambia [75, 76]. Research investigating the contribution of IQF pieces to dietary salt intake could influence the drafting of the next South African National Strategic Plan for the Prevention and Control of NCD, and provide an opportunity for policymakers to direct relevant action towards the broiler industry, given its role in consumers’ diets.

Similarly, the drive to improve affordability and consumption of broiler meat must be linked to the nutritional value and outcomes of the products consumed. The industry uses profits from high-value cuts and value-added products aimed at high-income consumers to support the provision of more affordable options, such as IQF pieces, to lower-income consumers. However, the processing of high-value cuts also generates trimmings that are sold as low-cost products, together with the broilers’ heads, feet, and intestines, which have little nutritional benefit. Whilst attempting to address affordability, nutritional equity is lost, with lower-income consumers denied access to healthier cuts because of price.

When considering the systems’ environmental aspects, industry stakeholders were aware of the impacts and threats of the climate crisis on their production systems. However, they provided little discussion around the industry’s own contribution to environmental degradation and climate change. Whilst most of the global environmental criticism of LDF is directed at ruminants, the impact of poultry production, via its dependence on concentrate feeds, should not be ignored, particularly in South Africa with its limited arable land, vulnerability to climate change, and population growth [9, 77]. The “fix that fails” archetype of intensive cereal production’s dependence on chemical inputs that ultimately undermines biodiversity, is of particular importance in the South African context. Given this apparent lack of awareness by stakeholders, further research and advocacy efforts are required to highlight the hidden costs of bringing commercial broiler meat to the plates of South African consumers. Furthermore, these environmental impacts should be scrutinised under the National Environmental Management Act, Principle 2 (4) (p), which states that those responsible for environmental damage and consequential adverse health impacts are liable for the costs of remedying them [78]. The system’s impact on land degradation and biodiversity loss through intensive feed production, its energy and water use in climate-controlled housing and in processing, the product packaging (and disposal), refrigeration of retailed products, and the disposal of waste and mortalities, require inclusion in any environmental impact discussion.

Support of the local feed value chain featured strongly in the PMP’s strategy, and in our discussions with broiler producers and feed manufacturers. South Africa is mostly self-sufficient in producing maize for livestock feed, but local soy production is supplemented with imports by up to 40% to meet demand [79, 80]. Imported soy is sourced almost entirely from Argentina, with a notable negative environmental impact [81]. The PMP’s strategy to expand local soy production for livestock feed will increase pressure and competition on land use for cereal production for a growing human population. However, policies that support the production of local soy, and regulate its environmental impact, would reduce the need for soy imports with its environmental footprint. In addition, an opportunity exists to expand the soy value chain for human food in parallel with livestock feed. This would lower the cost of raw ingredients for livestock feed, and offer a low-cost, soy-based protein source for human consumption as an alternative to low-cost (and low-nutritional value) broiler cuts.

Cross cutting challenges that face policymakers include the underlying socio-economic inequality that remains in South Africa, and to which the food system is not immune [82]. This study demonstrated this by highlighting the “success to the successful” archetype in both the Production and the Distribution modules, and that nutritional inequality is linked to affordability at the consumer level. The system underpinning inequality arguably has historic political roots, and is in urgent need of fundamental change to align it with the transformative SDG promise to “leave no one behind” [83]. The current plan for broiler system development continues to support commercial production, albeit with the caveat that commercial producers facilitate inclusion of more emerging famers as a means of addressing inequalities. However, this has the potential to further drive the “success to the successful” archetype and fails to acknowledge the contribution of small-scale and informal actors within the system. Their growth is undermined by the commercial system’s dominance and through the barriers this presents to them, such as access to inputs and markets [66]. The many benefits of small-scale village poultry systems that dominate much of sub-Saharan Africa are also ignored. These include household purchasing power (importantly for education and healthcare), household nutritional security, women empowerment, maternal and child health, and environmental sustainability, and the leverage that such systems offer to address several of the related SDGs [84].

A further system-wide challenge is to improve the coherence of food policy in South Africa. Institutional reform [29], together with a greater degree of industry consultation [85], have been called for to address this. Our discussions with broiler stakeholders identified gaps, and highlighted the urgent need for improvements in food policy coherence, particularly within food safety policies. Resolution of the protracted foodborne listeriosis outbreak in 2017–18 was hampered by government departments working in silos, private industry’s self-regulatory approach, and fragmented food safety policies [86]. Equally, a dichotomous laboratory system of poorly funded government institutions on one hand, and private, independent companies on the other, blocked the sharing of data, which would have hastened control of the outbreak [65]. In our policy discussions, several stakeholders identified the urgent need to control illegal imports and illegal processing activities that amplified FBD risk within the broiler system. They also recognised that food safety was a common goal for public and private stakeholders that could provide a leverage opportunity for public-private partnerships. However, the latter requires a foundation of trust, which had been lacking, but appears to have strengthened through the PMP agreement.

An overarching food safety agency that pulls together expertise from across government departments, industry, and academic disciplines is recommended. This could be responsible for food safety at the level of exports, imports, local production and processing, retail and food preparation, and consumption. Current food safety policies take a one-size-fits-all approach. However, in South Africa’s dualistic food system, the means to achieve compliance, within the food safety regulations, is out of reach for much of the informal sector. Although informal supply chains are the source of most food safety concerns in LMICs [6], their contribution to the supply of broiler meat is much lower in South Africa than in other LMICs, where the system may be less dominated by commercial production and formal markets. A risk-appropriate approach to food safety standards and regulations was suggested by stakeholders, to draw in small and informal actors who otherwise work below the enforcement level. Mitigation of food safety risk is closely related to resolving inequalities, especially for those living and working in resource poor settings, who are more likely to lack basic food safety knowledge, and access to electricity, refrigeration, and safe running water [25]. Therefore, support and education for informal market actors, and especially consumers, is recommended, since much of the FBD risk associated with broiler meat is due to improper handling, storage, and cross contamination of other uncooked foods [87, 88].

This study was limited by some stakeholders not responding to invitations to participate, which is not uncommon when approaching government departments and large-scale commercial institutions. In addition, the pressures of the COVID-19 pandemic (and ensuing lockdowns) on stakeholders’ work and personal lives may also have hampered willingness to engage during this period. The need for wider participation, to strengthen future stages of this work, is recognised. However, the limitation of participant numbers was somewhat overcome by the inclusion of a production industry representative, by the wider stakeholder participation achieved in the earlier research that underpinned the model development, and by the selection of individual stakeholders from different points in the value chain, and those with divergent interests within the system. This attempt to invite a wide range of stakeholders could have been strengthened further by initially conducting a formal stakeholder analysis to improve the identification of stakeholders and increase the inclusivity of this study.

Our research demonstrates the usefulness of a qualitative SD model to engage with a diverse range of stakeholders on the broader topics of nutrition, health, and environmental sustainability that surround the South African broiler industry. Similar systems-based analyses have been used to deepen stakeholders’ understanding of the system and identify trade-offs within India’s inequitable fruit and vegetable value chains [89], and climate-smart agricultural initiatives in Ghana [90]. Such systems modelling expands the value chain approach to integrate stakeholders perspectives, and illustrate linkages and feedbacks between interventions and broader nutritional outcomes [91]. Our approach facilitated discussion, beyond the short-term business strategies of powerful actors in the broiler value chain, to include consideration of the longer-term impacts on nutrition, health, and environmental sustainability that underpin several SDG objectives. This qualitative model with its modules also provides a foundation for researchers interested in developing parameterised models to simulate specific policy scenarios to yield quantitative results.

Group model-building workshops can be used to build a shared understanding of the system, and to reach consensus among stakeholders with respect to decisions and interventions [92, 93]. However, during preceding research [65], we identified the diverse range of opinions held by government, importers and local producers, and tension between them. When asked, most stakeholders preferred a non-workshop option for future engagement, and, hence, we opted to engage at the individual level. This approach, and the presentation of the model with its component modules, was well received by stakeholders. It helped facilitate discussions on policy gaps and ideas, and to identify areas for collaboration and capacity expansion within the system. It offered a safe environment for less powerful stakeholders to have an equal opportunity to express their concerns freely.

The modules and results provide a wider landscape in which policymakers can position the PMP and gain broader insights to direct future policies around it. The use of a food systems approach and SD modelling tools also provides a robust methodological reference for future food systems research with a policy focus. The methods used, such as the online questionnaire for problem statement consensus and to identify key system elements, inclusion of the seed model video in email invites, conducting virtual meetings on an individual or institutional basis, also provide options for researchers, who are constrained by travel restrictions, to engage with stakeholders remotely. Indeed, the use of such methods was congruent with the SHEFS programme’s commitment to environmental sustainability, which included using a virtual platform for biannual meetings (a decision made before COVID-19) [94].

Given the global surge in broiler meat production and consumption, this research provides transferrable lessons for policymakers in LMICs undergoing broiler development programs that face similar challenges to South Africa, such as competitive pressure from imports, foodborne disease vulnerability, growth of commercial producers at the cost of the smaller scale, climate change threats on natural resources and ecosystem services, and managing food security amidst the triple burden of malnutrition.

In addition, this research revealed similar issues within the wider LDF system in South Africa, particularly the beef and dairy systems. The consumption of beef and dairy products in South Africa are second only to broiler meat [64]. The production of both is rooted in intensive commercial systems, linked to formal markets, with the similar “success to the successful” archetypes creating exclusions and barriers to entry for non-commercial actors. Since there is less need for imported beef and dairy products, the threat of imports on the local producers is less. Therefore, there are fewer food safety issues relating to illegal imports of beef and dairy products, although the need for food safety policy coherence is equally applicable across the LDF system. Consumption of beef and dairy products have similar nutritional advantages and disadvantages to broiler meat, dependent on the type and volume of product consumed. Beef is the second most preferred ingredient for fast-food [39], whilst most yoghourts, and almost half the milk products, contain sugar and flavourings added during processing [95]. Beyond the publicised greenhouse gas emissions and environmental impact of ruminant production [96], dairy and intensive beef systems are also dependent on cereal concentrate feeds and fertilised pastures for grazing, which contribute to land use and biodiversity loss [3]. Their intensive production systems also require strict waste management to mitigate risks of contamination and eutrophication of water sources [97]. Therefore, additional research using a similar food systems approach is recommended to deepen policymakers’ understanding of the system-wide linkages within other LDF systems in South Africa.

## 5. Conclusion

A food systems approach and the use of SD tools provided a suitable platform to engage with commercial broiler system stakeholders, and facilitate discussions on policy areas. Current broiler policies in South Africa focus on supporting local production to increase consumption by providing an affordable LDF with a variety of product options. The qualitative SD model provided system-wide insights within the broader boundaries of nutrition, health, and environmental sustainability. Presenting a qualitative SD model in a modular format to stakeholders facilitated discussions to identify several additional issues for policymakers to consider. These included the dominance of large-scale actors within the system, the health impacts of increasing per capita consumption of products that are brined and a main component of fast-food, the challenge of making food safety policies more integrated and workable, and accounting for the full environmental footprint of the industry. Several policy considerations, specifically those relating to systemic inequality, are transferable to the LDF system in South Africa. Likewise, our methods may be suitable for use in research on similar broiler systems, undergoing rapid development in other LMIC settings. The food systems approach provided a wider perspective beyond the focus of food security. A broad systems-wide understanding is required as a foundation for policymakers to identify and engage with appropriate stakeholders from across the system, and work towards developing more integrated and transformative policies.

## Supporting information

S1 Text Online questionnaire. (DOCX)

S2 Text Seed model. (DOCX)

S1 Fig Qualitative SD model. (DOCX)
