# Supplementary material for: A food systems approach and qualitative system dynamics model to reveal policy issues within the commercial broiler chicken system in South Africa
Source: PLoS One. 2022 Jun 29;17(6):e0270756. doi: 10.1371/journal.pone.0270756 (PMC9242500; doi:10.1371/journal.pone.0270756)
Supplement: S1 Text — (DOCX) [file pone.0270756.s001.docx]

## S1. Online questionnaire

**The broiler meat system in South Africa**

**Section 1: Introduction**

Food systems are currently facing several important challenges in their mission to deliver healthy and nutritious food, in an environmentally sustainable and socially equitable manner. The Sustainable and Healthy Food Systems (SHEFS) programme aims to provide evidence for decision makers to address these challenges. Given the rapid rise in chicken consumption in South Africa, the associated broiler food system is the focus of our SHEFS research. Food systems are complex and are best understood and analysed using a "whole system" approach. Systems are defined as being comprised of elements or variables, which are interconnected and coherently organised to serve a function. Interconnections between elements may be through influence, feedback or actual physical flows. The system's elements/variables, their interconnections and their behaviour over time, form the foundation of a System Dynamics (SD) model. Through participation of key stakeholders (like yourself), with knowledge and expertise across various sectors within the system, we aim to create a systems map and develop a SD model of the broiler system, which can be used to generate evidence through simulating various policy scenarios. SD modelling starts with defining the problem and identifying key elements and variables within the system, and we would appreciate your participation in this process.

**Q1:** Through our recent interviews (with you and other key stakeholders) and from reviewing recent literature we propose the following as a Problem Definition: "The current broiler system in SA is under strain to produce sufficient food in an environmentally sustainable way, that is safe and nutritious, and meets the needs of a growing population in a socially equitable manner." Do you.....

(Please selected one of the following.)

- Agree
- partly agree
- disagree

If not in agreement, please give your reasons and offer suggestions or edits.

**Q2:** Over what time period has this problem, as defined (or redefined by you), existed?

(Please selected one of the following.)

- <5 years
- 5-10 years
- >10 years
- Don't know

**Section 2: System Elements/Variables**

Variables are key elements of a system, which may vary (increase or decrease) over time. For those unfamiliar with systems mapping, the simple example in the diagram below may be useful to demonstrate the process. A city council identifies a problem as “There is severe traffic congestion in our city.” If we consider what elements in the transport system are part of this problem, they may include those in the grey circles. If we then consider what influences each of these, then the elements in the blue boxes may be added. (Note: there may be influences between the original elements (Arrow A), and influencing variables may impact on more than one element. (Arrows B & C)


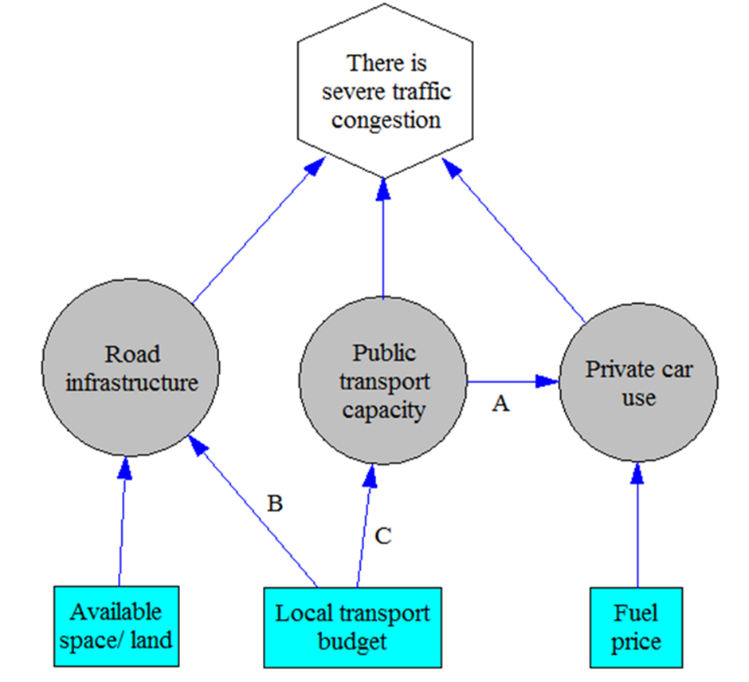


**Q3:** From our interviews, we identified key elements/variables associated with the defined problem as listed alphabetically below. Given your experience in the sector, please tick the elements, which you agree are associated with the problem as defined (or redefined by you). Use the "Others" option to add as many other elements that you think are associated with the problem. [Problem Definition: "The current broiler system in SA is under strain to produce sufficient food in an environmentally sustainable way, that is safe and nutritious, and meets the needs of a growing population in a socially equitable manner."]

(Please select any number of variables that apply.)

- Access to inputs for small-scale producers
- Access to markets for small-scale producers
- Broiler meat imports
- Capacity to implement legislation (You can specify which in next section)
- Feed availability/costs
- Food safety
- Food security
- Industry self-regulation
- Livelihoods of small-scale producers
- Natural resource availability
- Proportion of market held by vertically integrated companies
- Other…

**Section 3: Influencing factors**

**Q4:** Below you will find the same list of elements as in Section 2. For each one that you selected in Section 2, please use the free text space below to list the key factors that you think influence each, and briefly explain how. (E.g. from the traffic example above: Private car use: i) When the fuel price goes up, there is a decrease in private car use. ii) When the public transport capacity is poor, then there is an increase in private car use. iii) When there is a limited transport budget, road infrastructure and public transport capacity suffer.) Remember, you only need to give influencing factors for the ones you selected in Section 2. Click on the Back button at the bottom of the page if you need to remind yourself which ones you need to complete.

- Access to inputs for small-scale producers
- Access to markets for small-scale producers
- Broiler meat imports
- Capacity to implement legislation (You can specify which here)
- Feed availability/ costs
- Food safety
- Food security
- Industry self-regulation
- Livelihoods of small-scale producers
- Natural resource availability
- Proportion of market held by vertically integrated companies
- Other (Please use this space to list as many elements and their influencers as you wish.)

**Section 4: Data availability**

**Q5:** Please indicate if your institution collects and/or has access to data on any of the following? If yes, please indicate if and how the data can be shared for non-commercial purposes.

|  | Yes | Publicly available or free subscription | Paying subscription only | Potentially available on request | Not sharable | Not sure |
| --- | --- | --- | --- | --- | --- | --- |
| Prices (Market, import, or producer) |  |  |  |  |  |  |
| Quantities (produced, sold, distributed, imported) |  |  |  |  |  |  |
| Input quantities used (feed, water, electricity, medicines, day-old chicks, other) |  |  |  |  |  |  |
| Labour along supply chain (input production, integrators, small producers, processing or trading |  |  |  |  |  |  |
| Value additions or margins along the supply chain |  |  |  |  |  |  |
| Food safety/ animal health related surveillance data |  |  |  |  |  |  |
| Other (specify below) |  |  |  |  |  |  |

**Section 5**

Continued participation: We plan to work on building a model with ongoing participation and inputs from key stakeholders, such as yourself. The model will be used to simulate policy scenarios and provide evidence for decision makers. Your participation in this will be greatly appreciated and will be limited to ongoing communications through email, Skype, occasional meetings and/or a workshop. Your participation will help us ensure that the model has validity. It will also give you an opportunity help in directing future broiler system related policy, to ensure the production of healthy and nutritious food products in an environmentally sustainable, and socially equitable manner.

**Q6:** Are you willing to continue to be involved in this research over the next 6 months?

(Please choose 1 option)

Yes/No/Maybe

If Yes or Maybe, please indicate in what way(s)

- Email
- Skype
- 1 to 1 meetings
- group meetings/ workshops
- Other…
