# Supplementary material for: A food systems approach and qualitative system dynamics model to reveal policy issues within the commercial broiler chicken system in South Africa
Source: PLoS One. 2022 Jun 29;17(6):e0270756. doi: 10.1371/journal.pone.0270756 (PMC9242500; doi:10.1371/journal.pone.0270756)
Supplement: S2 Text — (DOCX) [file pone.0270756.s002.docx]

## S2. Seed Model

The seed model (Figure Sa) was developed and expanded from the online questionnaire elements and demonstrates linkages, feedback loops, archetypes and conceptual stocks and flows and follows standard labelling protocols for SD modelling as described by Sterman (2000). The market share held by commercial producers underpins their value-chain dominance via reinforcing loop R(i), which also strengthens their drive to self-regulation, further supporting their dominance via R(ii). Similarly, R(iii) and R(iv) illustrate that the commercial producers’ market share undermines growth of small-scale and emerging producers and their ability to overcome barriers to access (inputs and markets). These reinforcing loops form a key system archetype, described by Meadows (2009) as “success to the successful”. A deficiency in policy coherence and the capacity to implement it, is driving commercial industry self-regulation, and failing to resolve barriers for small-scale and emerging producers. Equally, these policy and capacity shortfalls have led to challenges to maintain food safety and has increased FBD risk. Production from both commercial and small-scale and emerging producers is influenced directly by natural resource availability and indirectly through its impact on feed price. Commercial producers are the main contributors to national production and are also most affected by imports that compete through similar formal outlets. R(v) illustrates the negative impact of increased imports on commercial production’s profitability, reducing the local production flow and stock, driving a higher import rate to meet demand.


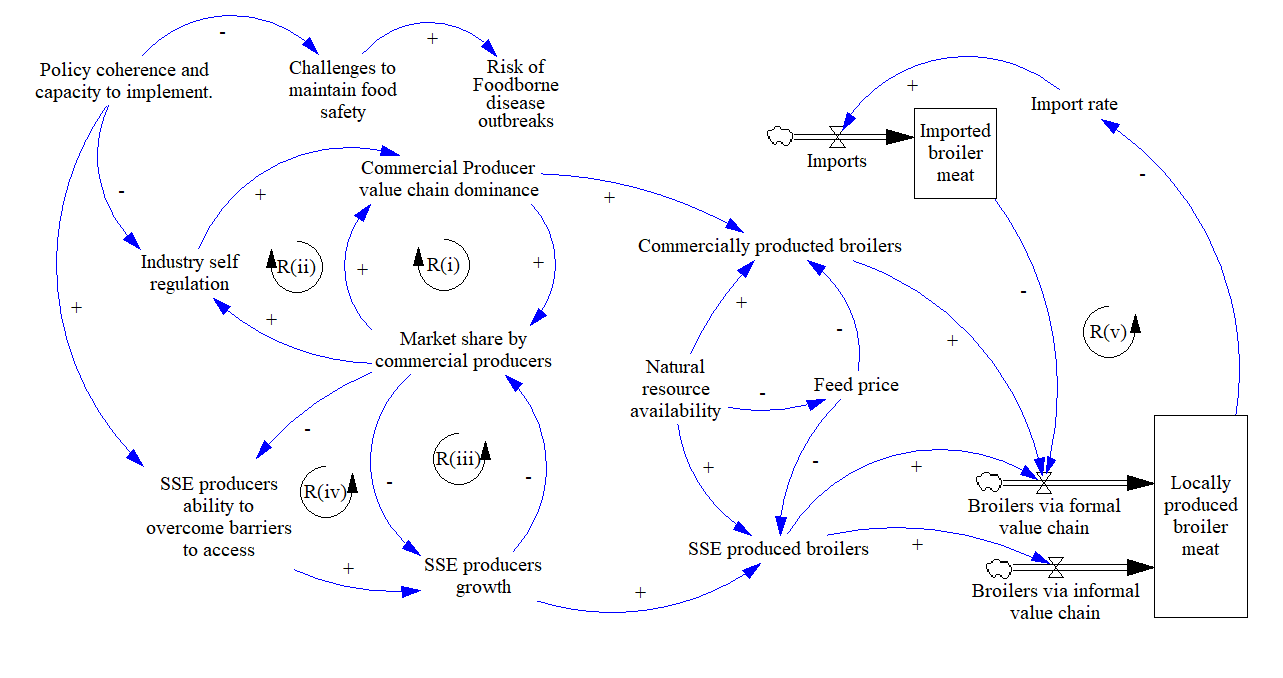


Figure S(a): Seed model of the South African broiler system. (Key: SSE= Small-scale and emerging. Standard SD model annotations used.)
