# Supplementary material for: A food systems approach and qualitative system dynamics model to reveal policy issues within the commercial broiler chicken system in South Africa
Source: PLoS One. 2022 Jun 29;17(6):e0270756. doi: 10.1371/journal.pone.0270756 (PMC9242500; doi:10.1371/journal.pone.0270756)
Supplement: S1 Fig — (DOCX) [file pone.0270756.s003.docx]

## S3. Qualitative SD model


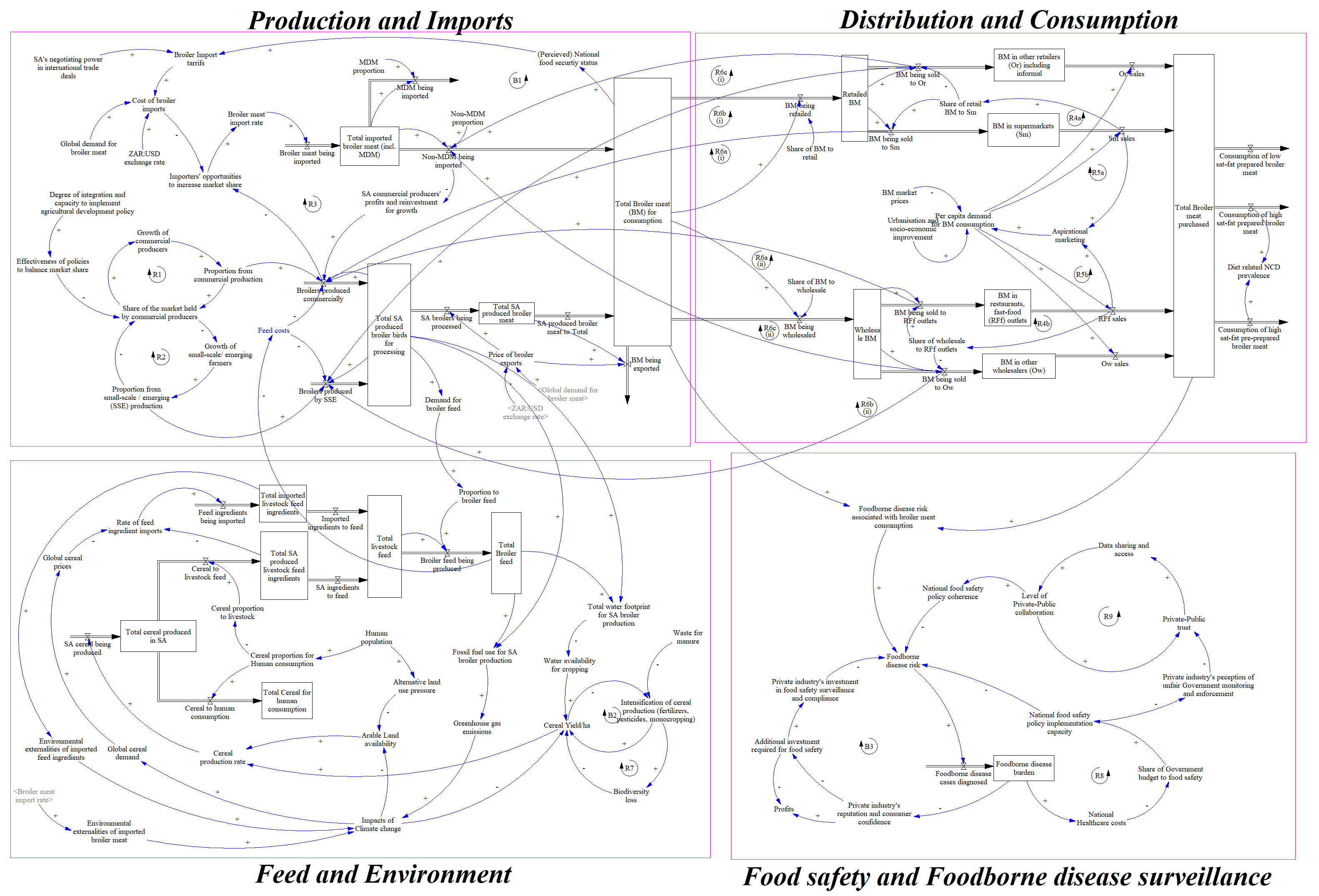


Figure S(b): Original whole qualitative SD model of the South African broiler system: a module within each of the four rectangles.
